# Enhancing Role Integrity for Peer Workers

**DOI:** 10.1007/s10597-023-01156-4

**Published:** 2023-07-04

**Authors:** Alex Elswick, Melinda Murdock, Amanda Fallin-Bennett

**Affiliations:** 1 Department of Family Sciences , University of Kentucky, Lexington, USA; 2 Voices of Hope-Lexington, Inc., Lexington, USA; 3 College of Nursing, University of Kentucky, Lexington, USA

**Keywords:** Peer support, Mutual aid, Peer work, Recovery support, Role integrity

## Abstract

Although informal peer support has been a central feature of recovery for people with substance use disorder (SUD), more recently there has been a stark increase in formal models of peer support. In the infancy of formalized peer support, researchers warned of potential threats to the integrity of the peer support role. Now, almost two decades into the rapid expansion of peer support, research has yet to evaluate the extent to which peer support is being implemented with fidelity and role integrity. The present study aimed to assess peer workers’ perceptions of peer role integrity. Qualitative interviews were conducted with 21 peer workers in Central Kentucky. Results suggest that the role of peers is not well understood by onboarding organizations, and thus, the integrity of peer support is diluted. Findings from this study suggest room for improvement in the training, supervision, and implementation of peer support.

Substance use and substance use disorder (SUD) are pervasive public health problems in the United States. In 2019, 60.1% of Americans 12 years or older used a substance (e.g. tobacco, alcohol, heroin, etc.) in the past month (SAMHSA, 2020a). According to the National Survey on Drug Use and Health, 7.7% (19.3 million) Americans have a SUD (SAMHSA, 2020b). In the last year and during the midst of the Covid-19 pandemic, overdose mortality reached a historic peak as 81,000 Americans died of overdose in a 12-month span (CDC, 2020). In addition to overdose mortality, SUD is associated with myriad health complications including heart disease, hypertension, Neonatal Abstinence Syndrome (NAS), and reduced quality of life (SAMHSA, 2020a).

The ever increasing burden associated with SUD has driven the need for new models of recovery support to fill the gaps in long-term addiction care (Kelly et al., 2017; McLellan et al., 2000). Accordingly, there has been a surge in the utilization of peer support in substance use and mental health settings (White, 2010). Peer workers, sometimes also referred to as “peer support specialists”, “peer mentors”, or “recovery coaches”, are people in recovery from SUD or a mental disorder who use their lived experience to provide non-professional, non-clinical recovery support services (Reif et al., 2014).

The concept of SUD peer support certainly is not novel. Peers in recovery have been long perceived as “wounded healers” with the potential to use their lived experience to help others in recovery (White, 2010, 2011). In fact, peer support forms the basis for mutual aid organizations and, as a recent Cochrane review indicates, informal peer support is likely the operative aspect of 12-step programs that make them efficacious (Kelly et al., 2020). However, the advent of the professional peer is a somewhat new application of peer support (Eddie et al., 2019) and research on peer support is still emerging. Existing research suggests peer support is associated with improvement in a host of substance use outcomes such as reduced days drinking (Bernstein et al., 2005), reduced odds of drinking to intoxication (Smelson et al., 2013), and reduced overall substance use (Mangrum, 2008; Rowe et al., 2007). Peer support also has a salutary effect on more global indicators of health such as improved treatment adherence (Tracy et al., 2011), improved treatment completion (Mangrum, 2008), decreased homelessness (Boisvert et al., 2008), decreased criminal justice involvement (Rowe et al., 2007), decreased hospitalizations (Kamon & Turner, 2013), and increased utilization of aftercare and mutual aid (Blondell et al., 2008). However, as multiple recent systematic reviews have pointed out, the existing research on peer support still emerging and thus relatively weak (Bassuk et al., 2016; Eddie et al., 2019). Most of the current research on peer workers lacks either comparison groups or experimental designs (Bassuk et al., 2016; Eddie et al., 2019). More importantly, existing research has yet to examine the extent to which peers are being implemented with integrity and fidelity to their core competencies.

Given the stark increase in peer utilization over the last decade, beginning with established Medicaid funding in 2007, peer workers are now a routine part of behavioral health service systems. Currently, one quarter of all behavioral health facilities in the U.S. offer peer services (e.g., Videka et al., 2019) and a majority of states have developed training and certification standards that have led to continued research, expansion of services, and a supported evidence base for these services (SAMHSA, 2015).

In the infancy of emergent peer support models, some researchers warned of external threats to the integrity of the peer worker role. William White (2006) identified the potential overlap among peer support with either addictions counseling or mutual aid sponsorship as potential sources of encroachment (White, 2006). “If it is to survive, a new service role must stake out its distinctive turf and justify its existence, and it must do so in the context of other roles claiming the same or adjoining territory” (p. 2). White explains that peer workers emerged in response to a need for a new type of de-professionalized peer role that fills gaps in the continuum of care which cannot be addressed by addiction professionals. Peer workers have more diffuse boundaries and are therefore better able to build rapport and provide close-contact, continuous recovery support. However, as peer workers are being implemented across the country with wide heterogeneity in terms of function within different organizational structures, the integrity of peer support is at risk. To support the evolving role of peer support, White recommended that future research consider further role definition and standards to ensure peer integrity as well as orientation, training, and supervision models.

Similarly, in 2012, SAMHSA held a roundtable discussion among early peer worker implementers to identify successes and challenges of peer support implementation. Participants identified the following as potential challenges: pressures to move toward a more clinical (professional) treatment model, misunderstandings and biases about peer workers, maintaining the recovery focus, as well as inadequate funding, infrastructure, and evidence for peer work (SAMHSA, 2012).

A similar evidence base has been concurrently developed to support the implementation of peer workers in mental health treatment settings, as well. Peer workers in mental health settings decrease psychotic symptoms, decrease depression, and increase engagement in self-care (Davidson et al., 2012). Notably, peer work in mental health faces similar threats to legitimacy and role clarity as peer work in SUD settings (Viking, et al., 2022). Despite these similarities, some differences exist in the role of peer work in these two settings (Chapman et al., 2018). Therefore, the scope of this paper focuses exclusively on peer workers in SUD treatment and recovery support settings.

Following both SAMHSA’s (2012) and White’s (2006) recommendations, the research aims in this study were (1) to describe the lived experience of peer workers, (2) to describe the tasks, roles, and functions of peer workers, (3) to assess peers’ perception of peer role integrity, and (4) to assess peers’ perception of peer worker training and supervision.

## Method

After obtaining informed consent, we conducted individual interviews with 21 peer worker participants in a private location within Voices of Hope, a recovery community center located in Central Kentucky. Individuals qualified for inclusion if they: (1) have worked as an SUD peer worker; (2) are at least 18 years of age; (3) can speak and read in English. Voices of Hope employees were excluded from this study. Participants were recruited via email or phone call from a Voices of Hope staff member as well as through the Voices of Hope monthly newsletter and Facebook page. Although Voices of Hope employs peer workers, Voices of Hope employees were excluded from this study. Interviews were conducted by experienced qualitative researchers (AE and AFB). Participants were compensated with a $25 gift card for their time.

The interviews were based on a semi-structured interview guide. The interviews were opened with the question, “What does ‘recovery’ mean to you?” To describe experiences as a peer worker, we asked questions such as: “Tell me about a typical day as a peer worker.” Example follow-up prompts were: “What does it feel like to work as a peer in a professional setting?” and “Tell me about the kinds of tasks you were asked to perform.” To assess shortcomings of peer support service provision, we asked questions such as: “Tell me about the biggest challenges you faced as a peer worker?” and “If you were in charge of peer support in our state, what would you do differently?”.

The interviews were voice recorded and professionally transcribed, and the transcripts were checked against the recordings for accuracy. A codebook was created by the research team after reviewing the transcripts and debriefing the data. Furthermore, additional codes were added based on the data as analysis progressed. Data were analyzed using content analysis in MAXQDA software. Transcripts were coded to consensus by trained members of the research team (AE and MM). Coded data were then organized into categories and themes. Throughout data analysis, the research team sought to maintain the thick, rick descriptions provided by participants in an effort to faithfully relay their experiences.

This qualitative study was approved by the University of Kentucky Institutional Review Board and performed in accordance with the ethical standards of research. The authors of this manuscript have no financial conflicts of interest to declare. However, Drs. Elswick and Fallin-Bennett are co-founders of Voices of Hope. Lastly, all authors certify responsibility for this manuscript.

## Results

### Sample Characteristics

Participants in this sample were mostly female (81.0%), mostly heterosexual (90.5%), overwhelmingly white (95.7%), and exclusively identified as non-Hispanic. The average age of participants was 40.3 years with an approximate time in recovery of 6.3 years.

Five themes emerged including: (1) Peers’ perception of their role; (2) Lack of training/preparedness by organizations onboarding peers; (3) Lack of appropriate supervision; (4) Disparity between peers’ role and tasks; (5) Tension between role as peer vs. professional.

### Peer Workers’ Perception of Their Role

Participants in this study agreed that a peer worker’s primary role is to use their lived experience to relate with clients and to advocate for their individual care. One participant reported “honestly, that’s what I think peer relationships are all about is hope. ‘Okay if you can do it, I can do it.’” Another participant elaborated to explain the reassurance clients experience when working with a peer “because she knows that I’ve experienced that brokenness…she knows that I have because she knows that I’ve been treated a different way because of my addiction.” Because of that shared experience, participants felt they were better equipped to build rapid rapport with clients. One participant explained “Like sitting in treatment, I related to my peer support specialist more than I related to my counselor who has some letters behind his name and you ain’t never shot dope in an alley and you ain’t never been locked up. You ain’t never kicked in a door. I’m gonna relate to this guy and I’m gonna get more from this guy.”

Participants also perceived their role as advocates not only for people in recovery but for recovery itself. One participant reported seeing peer workers as “ambassadors for recovery,” describing how peer workers are visible role models of recovery in the community at-large. However, the majority of participants in this study described their recovery advocacy on a micro or individual level. For instance, many participants reported experiences in which they found themselves at odds with their employer and on the side of advocating for an individual client. One participant who worked in a mental health court reported feeling as though it was the peer workers “against everybody else. Not that they’re opposed, they just don’t know the feeling or what can happen if you give somebody a chance. They just don’t know…Is she better off giving her a chance or just kicking her out on the street, you know?”.

### Lack of Training/Preparedness by Organizations

Although some participants requested additional training on navigating boundaries, peers generally reported feeling that their training was sufficient. One participant reported “Actually, training was pretty good. It covered just about everything. A lot of it was on ethics, what you should and shouldn’t do with the client.”

However, peers reported that the organizations that onboarded them did not understand the role of peers nor how to utilize them. When asked if their employer understands what they do, one participant replied “not really. When I got my job at [OTP] peer support was new. And it was just a part of their team that they started…and they didn’t really have a full understanding of what we did.” Other participants described their organization’s lack of understanding of the role of peers as “a shitshow” and “pulling ideas out of thin air.” Some participants experienced their employer’s lack of understanding of their role as a lack of respect for who they are and what they do. One peer reported “I wish they knew—some of them, I feel like they think it’s not a big deal if they had—they could care less if they had peer support, and there are some that do respect it. I wish there was more clinicians that had an idea of what peer support is and what they do, and maybe even a statistic-level thingy that would show that it does work.” Another participant echoed the notion that providers don’t understand the unique nature of their role, reporting “A lot of people don’t understand what I am there, because they’ve never had peer support there. And even when, you know, I’ll say, ‘I’m a peer, that’s my role in this’, they still don’t understand that. I’ll say ‘I’m in long-term recovery’, and sometimes they just don’t even understand that!”.

### Lack of Appropriate Supervision

Exacerbating the problem of employer’s not understanding the role of peer workers, participants further reported that their supervision was insufficient. First, many supervisors, like employers, were ill-prepared for peer support. When asked if she’d had supervision, one participant reported “I did have supervision, but it was a learning process for her as well because she had never had to do it, I don’t think.” Second, participants reported receiving inadequate supervision, if any at all. One peer reported “Yeah, we were—he sat down with us in the fourth month that we worked there, showed us a video on his laptop, and that was it. And, he documented every month that he was doing supervision with us, but he never actually did it.” Another participant reported a similar experience, saying “No. There was one time in the very beginning…But, she did not stay very long, and during that time when third peer support was employed there, he did that one-hour supervision with us, and I had asked him, ‘Aren’t you supposed to be doing supervision with us?’ ‘Oh yeah, I’ve already done it. You’re good. You don’t have to worry about it.’ I’m like, ‘Maybe I need it. You ever thought about that?’ ‘No, we’re good.’ So, it shocked me to actually have that where I’m working at now.” It should be noted that those participants who reported receiving adequate supervision indicated that it was a critical support for their job. Third, participants suggested that clinicians perform roles that are too dissimilar to peer workers to supervise appropriately. One participant explained “I just think that until you have done this job and you’ve worked within the parameters of where you fit in the treatment milieu, or whatever, spectrum, it’s hard to really supervise someone that’s trying to fit into that same square. You know what I’m saying?… And, I think people just go, ‘Ugh, you know. They’re fine. They can report to this person. They can report to this supervisor. They’re fine. It’ll all be good.’ But, it’s just not. It’s just different.” Instead, participants reported that “there should be senior peer support specialists that have been doing it for awhile” because “the best person to supervise a peer is a peer.”

### Disparity Between Peers’ Roles and Tasks

Peers reported that they were frequently asked to perform tasks that do not fit their prescribed role. When asked what tasks they performed as a peer worker, participants reported being asked to do “everything. Everything from doing the case management jobs, to doing the admin jobs, to being the janitor, to being the case manager, to peer support, literally everything. Asked to do everything with little pay, always the lowest person paid in the building, always asked to be the one to take out the trash, to pick up the cigarette butts outside while other people basically–weren’t doing anything. That’s kind of what we had to deal with.” Another participant reported being similarly treated as second-class in the workplace saying “Well, clinicians asked our peer support specialists to empty the trashcans when we first came on board.” Participants also reported being asked to perform duties that were not only outside their scope of practice, but more importantly, skirting an ethical boundary. One participant reported being asked to call in prescriptions with a DEA number. She replied “I’m not doing any of that. You need to find somebody in here to get that done because I’m in recovery and I don’t want my name—I’ve got enough hits on [the state’s Prescription Drug Monitoring Program].” On the whole, participants reported that the time they spend performing menial tasks unrelated to their role detracted from their ability to forge therapeutic alliances with clients.

### Tension Between Role as Peer vs. Professional

Participants identified an inherent dissonance in the role of peer workers who are tasked with being peers (non-professional, egalitarian) but also professionals (socialized to the workplace, expert posture). One participant explained how this tension in her role had consequences for her job evaluation. She reported “The biggest issue they have with me is professionalism—So, they teeter totter me. ‘Yes, you need to do your job. Yes, you need to be you. Yes, you need to share experience, strength, and hope. Now, you need to be a professional.’” Another participant agreed “I had never had a job in the corporate world, office setting, those kinds of things, so I really had to curb myself into that kind of lifestyle.” Finally, a participant pointed out that the title ‘peer specialist’ was inherently dissonant. She reported “And I hate the word specialist because I think that says I know something special about you that you don’t know, so I’m just gonna call it peer support. I get why they would call it specialist, but I also think—in the long run as far as terminology goes—it kind of goes against a lot of what we’re trying to do.”

## Discussion

The central finding of this study is that the role of peer workers is not well understood, except by peers themselves, presenting a threat to the integrity of the peer support role. Interestingly, peer workers in this study described their role almost verbatim to SAMHSA’s definition of peer support. For example, peers emphasized the importance of their work being “…all client-centered”, “listening more than talking”, or “helping to be a voice with and for [participants] “—consistent with SAMSHA core competencies of personalized client-centered support, advocacy, and collaborative relationships with peers (SAMHSA, 2015) and also consistent with existing qualitative research on peers’ experiences (Otte et al., 2020; Pantridge et al., 2016). Accordingly, peers in this study reported that their peer certification trainings adequately prepared them for their various roles.

In contrast, participants suggested the organizations which employed peers did not possess a working understanding of how peer support should be utilized. The results of this study suggest a lack of understanding that pervaded organizational structures from clinicians to supervisors to administrators. As a result, onboarding organizations lacked sufficient training, supervision, preparedness, and role definition for peers within the workplace. These findings are consistent with existing research which suggests peer workers face inadequate infrastructure and supervision as well as misunderstandings about their role (SAMHSA, 2012).

Although peers in this study received training for their role, none were aware of any training undergone by their employers. This suggests a lack of consistency and/or transparency in supervisor training. The resultant disparity in role understanding led to conflicts in the workplace. Namely, peers reported feeling pressured to code switch between the expectation of professionalism from their employers and the egalitarian, de-professionalized role for which their training and experience had prepared them. This, too, is a finding consistent with existing literature on external pressures to professionalize the peer role. SAMHSA’s stakeholder roundtable found that, particularly in clinical settings, “peer leaders risk being views as ‘junior counselors’ or ‘counselors light’ and losing the authenticity of [peer support] (SAMHSA, 2015, p. 13). Not only does this disparity in role and task undermine the service role of peer support, but it also places undue workplace pressure upon peer workers who are typically early in their recovery and thus vulnerable to psychosocial stress (Tate et al., 2008; White, 2006).

Lack of preparedness by onboarding organizations is potentially harmful to peers themselves but also represents a threat to the integrity and future of peer support more broadly. First, with insufficient understanding of the peer role, employers may be implementing peer workers sub-optimally. Consequently, clients may not receive the best possible care. Second, and more importantly, the misapplication of peer support represents a real and meaningful threat to the integrity and future of peer support.

Role integrity is consistency between one’s role and tasks, despite the fact that the role may manifest differently (Stratford et al., 2017). Other mental health providers can maintain role integrity across work settings because there is infrastructure already in place to support their positions, whereas there is a decided lack of infrastructure to support peer support (SAMHSA, 2012). Peer workers essential role in the treatment process becomes trivialized when employers attempt to utilize them in ways they were never intended defined by SAMHSA. This “watering down” of the peer support role and the use of peers as a “jack of all trades” does not render them more potent but less so. Further, as peers are tasked with jobs that do not align with their purpose, role ambiguity and confusion by employers lead to attempts to professionalize peers and assimilate them to other provider roles on the treatment team. Professionalism poses a threat to peer workers who are intended to be de-professionalized in order to form egalitarian relationships with clients. As the peer role becomes professionalized, peer workers are pulled away from the practice of using lived experience, which makes them effective at building rapport (Adams, 2020; SAMHSA, 2012). Ultimately, the misuse of, and harm to, peers lead to poor outcomes in services which produce inaccurate evaluations of peer support effectiveness. Employers and health organizations cannot base the efficacy of the peer support model on their execution of janitorial duties or case management and administrative tasks. Peer workers deserve to be evaluated and supported on their ability to build rapport and use their related experiences to guide and support clients through the treatment process. And further, it is the responsibility of the employer and organizations to create a workforce structure that is conducive to this work and that teaches peers expectations about workplace culture. When peer workers are utilized as intended, peers, clients, and organizations mutually benefit (Bassuk et al., 2016; Reif et al., 2014). Under ideal circumstances, peers would be able to do meaningful and fulfilling work that aligns with their role, identity, and strengths. Clients would receive the support and guidance they need from peers during the recovery process, via trusting relationships based on mutual experiences. And finally, treatment facilities would see the positive patient mental health and recovery outcomes they expect.

To improve the implementation of peer support, numerous improvements should be considered. First, onboarding organizations must be better prepared to implement peer support. This requires the development of trainings to clarify for employers the intended role of peer support as well as how the peer role differs from counselors and sponsors. Such trainings could explain how peers should be utilized, the tasks for which they are trained, and the standards by which their performance should be evaluated. In addition, this training could explain to employers that peers are deliberately de-professionalized and should be supported and evaluated accordingly. Second, peer supervision may be more effectively delivered by “senior” peers who have experience in the role that they are evaluating. They may be better equipped than social workers and other credentialed practitioners to monitor and evaluate the peer role because they have their own experience in a peer role. Third, per the peers in this study, we recommend reconsidering the title “peer support specialist” as it positions peers in a top-down, expert or specialist stance. The titles “peer worker”, “peer support”, “recovery coach”, etc. better reflect the nature of the peer role.

## Limitations

This study is not without limitations. First, as with all qualitative research, there is the potential for bias. In an effort to address this potential limitation, multiple validation strategies were incorporated including using thick, rich description and bracketing lived experience (Creswell, 2006). Second, participants were recruited for this study through Facebook and email communications from a recovery community organization in Central Kentucky. Thus, the peers in this sample likely represent a more homogenous sample than peer workers writ large. Also, the sample in this study was not diverse, which limits the generalizability of results.

## Conclusion

Substance use and SUD continue to levy a heavy burden on society and peer support provides an emerging recovery support pathway. As the utilization of peer support expands throughout behavioral health systems, research is needed to assess how peer workers are being used in the field. The results of this qualitative study suggest considerable opportunities to improve training, supervision, and infrastructure to support the peer worker role. Further research is needed to enhance the effectiveness of peer support but also to protect its integrity.

## References

[CR1] Adams WE (2020). Unintended consequences of institutionalizing peer support work in mental healthcare. Social Science & Medicine.

[CR2] Bassuk EL, Hanson J, Greene RN, Richard M, Laudet A (2016). Peer-delivered recovery support services for addictions in the United States: A systematic review. Journal of Substance Abuse Treatment.

[CR3] Bernstein J, Bernstein E, Tassiopoulos K, Heeren T, Levenson S, Hingson R (2005). Brief motivational intervention at a clinic visit reduces cocaine and heroin use. Drug and Alcohol Dependence.

[CR4] Blondell RD, Behrens T, Smith SJ, Greene BJ, Servoss TJ (2008). Peer support during inpatient detoxification and aftercare outcomes. Addictive Disorders & Their Treatment.

[CR5] Boisvert RA, Martin LM, Grosek M, Clarie AJ (2008). Effectiveness of a peer-support community in addiction recovery: Participation as intervention. Occupational Therapy International.

[CR6] CDC. (2020). Overdose deaths accelerating during COVID-19. Retrieved from https://www.cdc.gov/media/releases/2020/p1218-overdose-deaths-covid-19.html

[CR7] Chapman S, Blash L, Mayer K, Spetz J (2018). Emerging roles for peer providers in mental health and substance use disorders. American Journal of Preventive Medicine.

[CR8] Creswell, J. W. (2006). *Qualitative Inquiry and Research Design: Choosing among Five Approaches [with CD-ROM]. (*Second Edition: SAGE Publications (CA)

[CR9] Davidson L, Bellamy C, Guy K, Miller R (2012). Peer support amon persons with severe mental illnesses: A review of evidence and experience. World Psychiatry.

[CR10] Eddie D, Hoffman L, Vilsaint C, Abry A, Bergman B, Hoeppner B, Kelly JF (2019). Lived experience in new models of care for substance use disorder: A systematic review of peer recovery support services and recovery coaching. Frontiers in Psychology.

[CR11] Kamon, J., & Turner, W. (2013). *Recovery coaching in recovery centers: What the intial data suggest: A brief report from the Vermont Recovery Network*. Retrieved from Montpelier, VT

[CR12] Kelly JF, Abry A, Ferri M, Humphreys K (2020). Alcoholics anonymous and 12-step facilitation treatments for alcohol use disorder: A distillation of a 2020 cochrane review for clinicians and policy makers. Alcohol & Alcoholism.

[CR13] Kelly JF, Bergman B, Hoeppner BB, Vilsaint C, White WL (2017). Prevalence and pathways of recovery from drug and alcohol problems in the United States population: Implications for practice, research, and policy. Drug and Alcohol Dependence.

[CR14] Mangrum. (2008). *Creating access to recovery through drug courts: Final evaluation report*. Retrieved from Austin, TX

[CR15] McLellan AT, Lewis DC, O'Brien CP, Kleber HD (2000). Drug dependence, a chronic medical illness: Implications for treatment, insurance, and outcomes evaluation. JAMA.

[CR16] Otte I, Werning A, Nossek A, Vollmann J, Juckel G, Gather J (2020). Beneficial effects of peer support in psychiatric hospitals. A critical reflection on the results of a qualitative interview and focus group study. Journal of Mental Health.

[CR17] Pantridge CE, Charles VA, DeHart DD, Iachini AL, Seay KD, Clone S, Browne T (2016). A qualitative study of the role of peer support specialists in substance use disorder treatment: Examining the types of support provided. Alcoholism Treatment Quarterly.

[CR18] Reif S, Braude L, Lyman DR, Dougherty RH, Daniels AS, Ghose SS, Delphin-Rittmon ME (2014). Peer recovery support for individuals with substance use disorders: assessing the evidence. Psychiatric Services.

[CR19] Rowe M, Bellamy C, Baranoski M, Wieland M, O'Connell MJ, Benedict P, Sells D (2007). A peer-support, group intervention to reduce substance use and criminality among persons with severe mental illness. Psychiatric Services.

[CR20] SAMHSA. (2012). Retrieved from Rockville, MD: http://www.williamwhitepapers.com/pr/CSAT%20Perspectices%20on%20Peer%20Recovery%20Support%20Services%202013.pdf

[CR21] SAMHSA. (2015). Core competencies for peer workers in behavioral health services. Retrieved from https://www.samhsa.gov/sites/default/files/programs_campaigns/brss_tacs/core-competencies_508_12_13_18.pdf

[CR22] SAMHSA. (2020a). *National Survey on Drug Use and Health*. Retrieved from Rockville, MD: https://www.samhsa.gov/data/sites/default/files/reports/rpt29393/2019NSDUHFFRPDFWHTML/2019NSDUHFFR1PDFW090120.pdf

[CR23] SAMHSA. (2020b). The National Survey on Drug Use and Health: 2019. Retrieved from https://www.samhsa.gov/data/sites/default/files/reports/rpt29392/Assistant-Secretary-nsduh2019_presentation/Assistant-Secretary-nsduh2019_presentation.pdf

[CR24] Smelson DA, Kline A, Kuhn J, Rodrigues S, O'Connor K, Fisher W, Kane V (2013). A wraparound treatment engagement intervention for homeless veterans with co-occurring disorders. Psychological Services.

[CR25] Stratford, A., Halpin, M., Phillips, K., Skerritt, F., Beales, A., Cheng, V., Hammond, M., O-Hagan, M., Loreto, C., Tiengtom, K., Kobe, B., Harrington, S., Fisher, D., & Davidson, L. (2017). The growth of peer support: An international charter. *Journal of Mental Health, **28*(6), 627-632. 10.1080/09638237.2017.1340593 28682640

[CR26] Tate SR, Wu J, McQuaid JR, Cummins K, Shriver C, Krenek M, Brown SA (2008). Comorbidity of substance dependence and depression: Role of life stress and self-efficacy in sustaining abstinence. Psychology of Addictive Behaviors : Journal of the Society of Psychologists in Addictive Behaviors.

[CR27] Tracy K, Burton M, Nich C, Rounsaville B (2011). Utilizing peer mentorship to engage high recidivism substance-abusing patients in treatment. American Journal of Drug & Alcohol Abuse.

[CR28] Videka, L., Neale, J., Page, C., Buche, J., Beck, A., Wayment, C. & Gaiser, M. (2019). National analysis of peer support providers: Practice settings, requirements, roles, and reimbursement. *Behavioral Health Workforce*

[CR29] Viking T, Wenzer J, Hylin U, Nilsson L (2022). Peer support workers' role and expertise and interprofessional learning in mental health care: A scoping review. Journal of Interprofessinal Care.

[CR30] White, W. L. (2006). *Sponsor, recovery coach, addiction counselor: The importance of role clarity and role integrity*. Retrieved from Philadelphia, PA: https://facesandvoicesofrecovery.org/wp-content/uploads/2019/06/The-Importance-of-Role-Clarity-and-Role-Integrity.pdf

[CR31] White, W. L. (2010). Nonclinical addiction recovery support services: History, rationale, models, potentials, and pitfalls. *Alcoholism Treatment Quarterly, 28*(3), 256–272. Retrieved from http://ezproxy.uky.edu/login?url=http://search.ebscohost.com/login.aspx?direct=true&db=swh&AN=81115&site=ehost-live&scope=site 10.1080/07347324.2010.511057 PMC6419765 30880870

[CR32] White WL (2011). Wounded healers in recovery. Alcoholism Treatment Quarterly.

